# Multimodal Data Integration Reveals Mode of Delivery and Snack Consumption Outrank Salivary Microbiome in Association With Caries Outcome in Thai Children

**DOI:** 10.3389/fcimb.2022.881899

**Published:** 2022-05-23

**Authors:** Tong Tong Wu, Jin Xiao, Samantha Manning, Prakaimuk Saraithong, Komkham Pattanaporn, Bruce J. Paster, Tsute Chen, Shruti Vasani, Christie Gilbert, Yan Zeng, Yihong Li

**Affiliations:** ^1^ Department of Biostatistics and Computational Biology, University of Rochester Medical Center, Rochester, NY, United States; ^2^ Eastman Institute for Oral Health, University of Rochester Medical Center, Rochester, NY, United States; ^3^ Department of Internal Medicine, Division of Infectious Diseases, Medical School University of Michigan, Ann Arbor, MI, United States; ^4^ Mae Fah Luang University School of Dentistry, Chiang Rai, Thailand; ^5^ Department of Microbiology, Forsyth Institute, Cambridge, MA, United States; ^6^ Department of Microbiology and Immunology, University of Rochester Medical Center, Rochester, NY, United States; ^7^ Department of Public and Ecosystem Health, Cornell University Master of Public Health Program, Ithaca, NY, United States

**Keywords:** oral microbiome, saliva, multimodal analysis, early childhood caries, machine learning, diet

## Abstract

Early childhood caries (ECC) is not only the most common chronic childhood disease but also disproportionately affects underserved populations. Of those, children living in Thailand have been found to have high rates of ECC and severe ECC. Frequently, the cause of ECC is blamed on a handful of cariogenic organisms, such as *Streptococcus mutans* and *Streptococcus sobrinus*. However, ECC is a multifactorial disease that results from an ecological shift in the oral cavity from a neutral pH (~7.5) to an acidic pH (<5.5) environment influenced by the host individual’s biological, socio-behavioral, and lifestyle factors. Currently, there is a lack of understanding of how risk factors at various levels influence the oral health of children at risk. We applied a statistical machine learning approach for multimodal data integration (parallel and hierarchical) to identify caries-related multiplatform factors in a large cohort of mother-child dyads living in Chiang Mai, Thailand (N=177). Whole saliva (1 mL) was collected from each individual for DNA extraction and 16S rRNA sequencing. A set of maternal and early childhood factors were included in the data analysis. Significantly, vaginal delivery, preterm birth, and frequent sugary snacking were found to increase the risk for ECC. The salivary microbial diversity was significantly different in children with ECC or without ECC. Results of linear discriminant analysis effect size (LEfSe) analysis of the microbial community demonstrated that *S. mutans*, *Prevotella histicola*, and *Leptotrichia hongkongensis* were significantly enriched in ECC children. Whereas *Fusobacterium periodonticum* was less abundant among caries-free children, suggesting its potential to be a candidate biomarker for good oral health. Based on the multimodal data integration and statistical machine learning models, the study revealed that the mode of delivery and snack consumption outrank salivary microbiome in predicting ECC in Thai children. The biological and behavioral factors may play significant roles in the microbial pathobiology of ECC and warrant further investigation.

## Introduction

Early childhood caries (ECC) is the single most common chronic childhood disease, known to disproportionately afflict more than 73% of underprivileged preschool children worldwide (Dye et al., 2007; Dye et al., 2012).

More than 50% of 3-year-old Thai children experienced ECC (Bureau of Dental Health, 2018). Chronically persistent ECC can progress to severe-ECC (S-ECC) impacting the primary dentition and adding a significant financial burden to the involved families. Several studies have depicted a higher prevalence of ECC in Thailand, with as high as 44.5% non-cavitated initial lesions and 24.5% cavitated advanced lesions being reported among 15–19-month-old children in Central Thailand (Ledder et al., 2018). In addition, a prospective study demonstrated ECC progressed rapidly among 9-18-month-old children in Southern Thailand (reported incidence of 2% at nine months, 22.8% at 12 months, and 68.1% at 18 months of age) (Thitasomakul et al., 2006). Nationwide, the ECC prevalence among 3-year-olds and 5-year-olds was 52.9% and 75.6%, respectively; 98% of them had untreated caries (Bureau of Dental Health, 2018). This highlights the urgent need to raise awareness among Thai parents/caregivers and healthcare providers to implement more effective preventive strategies for ECC control.

While ECC is an infectious disease initiated by cariogenic pathogens, it is now understood as a multifactorial and ecology-based disease (Simon-Soro and Mira, 2015), with the interplay between host, environment, and oral microbiota affecting the onset and severity of the disease (Pitts et al., 2017; Bowen et al., 2018). Factors such as environmental (exposure to water fluoridation and fluoride toothpaste, oral hygiene), biological (dental plaque accumulation, cariogenic microbial composition), lifestyle (breastfeeding pattern, frequent sugar consumption, obesity), and socio-cultural (low socio-economic status, marital status, maternal oral health perception, utilization of dental care) have been linked to ECC (Chanpum et al., 2020). A longitudinal cohort study among 3-year-old children in Northern Thailand reported that suboptimal water fluoridation, low socioeconomic status, frequent sugar consumption, and dental plaque accumulation contributed to high ECC prevalence (44.1%) (Peltzer and Mongkolchati, 2015). Additionally, studies have reported inconclusive results for the association between ECC and prolonged breastfeeding habits. However, one cross-sectional study indicated that ECC’s higher prevalence and severity among breastfed Thai children was significantly associated with factors such as age, children’s oral health status, and breastfeeding pattern/duration (Chanpum et al., 2020). Furthermore, our previous study among 182 3-year-old and 166 5-year-old Thailand preschool children revealed that mode of birth delivery was significantly correlated with *Streptococcus mutans* colonization and caries outcomes in young Thai children (Pattanaporn et al., 2013; Saraithong et al., 2015). Taken together, findings from these studies confirm that ECC is a public health concern in Thailand owing to the rising disease burden. With a thorough understanding of the underlying risk factors and effective prevention strategies, it is hoped that this knowledge will help reduce the ECC disease burden among Thai children as well as children worldwide.

Due to the multifactorial etiology of ECC, a valid prediction model that utilizes sensitive microbial markers from oral microbiota (including but not limited to *S. mutans*) at early life, together with medical-socio-behavior and environmental factors, would offer a substantial opportunity to predict ECC and help generate a personalized preventive regimen. A few recent studies have demonstrated machine learning predictive modeling using 16S rRNA sequencing of oral samples, but they lack consideration for multifactorial nature of tooth decay (Teng et al., 2015; Grier et al., 2020). Our team has recently developed a multivariate ECC prediction model that uses a machine learning approach incorporating microorganism composition and demographic-environmental factors among a small group of US mother-child dyads (Wu et al., 2021). Here, we further developed stepwise, parallel integrative, statistical machine learning (ML) models to identify caries-related multiplatform factors [environment, biomedical (child and maternal), socio-behavior-feeding, and oral microorganisms) in a large Thai mother-child dyad.

## Methods

### Study Population

This cross-sectionally-designed study is a subset of a parent study detailed previously (Pattanaporn et al., 2013). The current study enrolled 177 3-year-old children and their biological mothers during the children’s immunization visit at the Health Promoting Hospital in Chiang Mai, Thailand, from July to December of 2009. Children who had significant congenital anomalies, chronic illness, or had taken any antibiotics within six weeks prior to examinations were excluded. A structured questionnaire was used to obtain data on family socio-demographics (age, sex, primary care provider, and maternal/family background), maternal pregnancy and past medical history (mode of delivery, gestational age, birth weight), as well as child’s feeding practice (breastfeeding, bottle feeding, and mother pre-chewing food), dietary habits (consumption of fruit juice, snacks, gum, lollipop candy, dried fruit, soft drink, child sleeping with bottle), and oral health practices. Information about delivery methods, gestational age, and child’s birth weight were further verified from accessing the hospital medical records. Chi-squared statistics were used to examine the differences between categorical variables. The Ethical Committee approved the protocol for this study of the Faculty of Dentistry, Chiang Mai University, Thailand (No. 12/2008). Written informed consent was obtained from all mothers or responsible caregivers at the time of children’s hospital visits.

### Dental Examination and Saliva Collection

Two calibrated dentists performed a comprehensive dental examination for all children and their mothers using the WHO criteria of decayed, missing, and filled teeth (DMFT for the mothers and *dmft* for the children) (Organization. WH, 1997). The presence of ECC was also recorded as a detectable white-spot lesion or cavity following American Academy of Pediatric Dentistry criteria (American Academy of Pediatric Dentistry, 2022). Approximately 1 ml whole saliva was collected from the mothers and their children after chewing a piece of paraffin wax for 1 min under the close supervision of a medical professional. The saliva samples were stored in centrifuge tubes and immediately transferred to a -20°C freezer in the microbiology laboratory at the Chiang Mai University Faculty of Dentistry until further microbiome analysis.

### DNA Extraction and 16S rRNA Sequencing

The microbial DNA extractions were performed at the microbiology laboratory at New York University College of Dentistry using a standard procedure (Saraithong et al., 2015). Briefly, 500µl of each saliva sample was used for whole-genome DNA extraction using Epicentre MasterPure™ DNA Purification kit. The 16S rRNA sequencing was conducted at the Forsyth Institute, Cambridge, MA. A total of 129 genus-specific probes and 638 species-level probes were used for bacterial identification. QIIME 1.9.1 (Caporaso et al., 2010) was used to quantify the composition and diversity of each community based on its open-reference OTU picking facility. Sequencing data that passed quality controls were included in this study to develop a caries prediction model and were assigned to operational taxonomic units (OTUs). OTUs having zero counts across all the samples or only appearing in one sample were removed from further analysis.

Alpha diversity analysis was performed using the phyloseq package (McMurdie and Holmes, 2013). The results were plotted across samples and reviewed as box plots for each group or experimental factor. Further, the statistical significance of grouping based on experimental factor was also estimated using a t-test. Beta-diversity similarity or distance between sample was measured using non-phylogenetic Bray-Curtis distance. Ordination-based methods Principal Coordinate Analysis (PCoA) was used to visualize these matrices in the 2D plot where each point represents the entire microbiome of a single sample. The statistical significance of the clustering pattern in ordination plots for beta diversity was evaluated using anyone among Permutational MANOVA (PERMANOVA). A *P* value less than 0.05 was considered statistically significant.

The Core microbiome analysis was adopted from the core function in the R package microbiome. The result of this analysis was represented in the form of a heatmap of core taxa or features where Y-axis represents the prevalence level of core features across the detection threshold (Relative abundance) range on the X-axis. A heat tree map depicting the OTU classifications and differential abundance comparison between salivary microbiome of children with ECC and caries-free children was employed. Taxa that have a significant abundance difference between ECC children vs. caries-free children was measured by the Wilcoxon Rank Sum test. Linear discriminant analysis (LDA) coupled with effect size (LEfSe) analysis (Segata et al., 2011) and non-parametric factorial Kruskal-Wallis sum-rank test were used to identify differentially abundant bacterial taxa regarding ECC status. Bacterial features were considered to be significant based on the logarithmic LDA score threshold >2.0 and false discovery rate <0.1.

### Development of Machine Learning Prediction Models

The primary outcome was the caries status (Y/N) of children. The independent variables were grouped into three platforms based on the distal to proximal relationship to ECC: 1) Maternal socio-demographic-behavior-environmental factors (seven variables); 2) Children’s socio-demographic-behavior-environmental factors (20 variables); 3) Children’s oral microbial factors (386 variables). The characteristics of the two groups (ECC and Caries-free children) were compared using t-test for continuous data and Chi-square or Fisher’s exact tests for categorical data.

OTUs with fewer than 10 reads were removed from the dataset to ensure the sufficient depths. The centered log-ratio (CLR) transformation was applied to the relative abundance of taxa, where for each subject, the sample vector undergoes a transformation based on the logarithm of the ratio between the individual elements and the geometric mean of the vector. CLR removes the value-range restriction of percentages (relative abundance is a percentage) but keeps the sum constraint of compositional data.

First, we took the parallel integration approach to integrate data from different platforms. Data collected on the three platforms were put together and fitted in penalized logistic regression model using least absolute shrinkage and selection operator (LASSO) with the response variable being the binary indicator of whether the subject has caries or not (1 = caries present, 0 = no caries present). The LASSO tuning parameter was chosen using k-fold (k = 10) cross-validation. The solution path was created to show the order of the variables entering the model.

Then, the hierarchical integration approach, a two-step procedure, was used to integrate data collected on the three platforms listed above. First, a LASSO penalized logistic regression model was fit for each of the four platforms, with the same response variable of having caries or not. Next, the LASSO tuning parameter was chosen using k-fold (k = 10) cross-validation for each model. The set of variables selected for each of the four platforms were then collected together and used as candidate variables to fit the final LASSO-penalized logistic regression model. Again, 10-fold cross-validation was used to determine the optimal number of variables in the final model. Finally, the solution paths for the four models and the final model were created.

## Results

The demographic characteristics of the children with or without ECC are shown in **Table 1**. No significant differences were found between the ECC and caries-free children regarding maternal age and antibiotic use. There was an increase of ECC in mothers who prechewed food for their children, although the difference was marginally significant (p=0.051). In terms of children’s factors, significantly more ECC children than caries-free children were born vaginally [63.27% vs. 43.04%, p=0.007; Odd’s ratio = 2.28, 95% CI (1.19, 4.37)] or preterm (45.95% vs. 30.38%, p=0.035). More than 83% ECC children were colonized with high levels of *S. mutans* compared to 20% in caries-free children (p<0.001). More caries-free children were found to use fluoridated toothpaste (p=0.009); were bottle fed (p=0.040); and less frequently consumed soft drinks (p=0.003), snacks (p=0.003), lollipop (p=0.017), and other candies (p=0.015).

**Table 1:** Characteristics of Thai children with and without ECC.

Independent covariates	ECC children (n = 98)	Caries-free children (n = 79)	P value
**Maternal factors**			
**Mothers biomedical**			
Age group			
20 year old	41.84% (41)	27.85% (22)	0.053
30 year old	58.16% (57)	72.15% (57)	
Antibiotic use (Y)	88.78% (87)	84.81% (67)	0.435
**Maternal behavior**			
Caregiver			
Mother (Full time)	53.06% (52)	43.04% (34)	0.406
Mother (Not full time)	36.73% (36)	45.57% (36)	
Other than mother	10.20% (10)	11.39% (9)	
Mother prechewing food	37.76% (37)	24.05% (19)	0.051
Smoking	2.04% (2)	1.27% (1)	0.581
**Maternal oral health**			
Caries (Y)	94.90% (93)	92.41% (73)	0.495
* S. mutans* level			
SM Strip score 0 - CFU <10^4^	32.65% (32)	35.44% (28)	0.408
SM strip score 1 – CFU 10^4^ - 10^5^	31.63% (31)	37.97% (30)	
SM strip score 2 – CFU 10^5^ - 10^6^	25.51% (25)	15.19% (12)	
SM strip score 3 – CFU >10^6^	10.20% (10)	11.39% (9)	
			
**Children’s factors**			
**Child biomedical**			
Age group			
2 years old	34.69% (34)	29.11% (23)	0.430
3 years old	65.31% (64)	70.89% (56)	
Sex (Male)	62.24% (61)	56.96% (45)	0.476
Delivery mode			
Vaginal	63.27% (62)	43.04% (34)	0.007*
C-section	36.73% (36)	56.96% (45)	
Preterm birth (Y)	45.92% (45)	30.38% (24)	0.035*
Low birth weight (Y)	3.06% (3)	5.06% (4)	0.382
Antibiotic use (Y)	88.78% (87)	94.94% (75)	0.116
**Child oral health behavior**			
Brush teeth daily (Y)	87.76% (86)	93.67% (74)	0.184
Uses fluoridated toothpaste (Y)	57.14% (56)	75.95% (60)	0.009*
**Child eating habits**			
Soft drink			
Never/Rarely	21.43% (21)	44.30% (35)	0.003*
Sometimes	21.43% (21)	10.13% (8)	
≥1 per day	57.14% (56)	45.57% (36)	
Fruit Juice			
Never/rare	10.20% (10)	13.92% (11)	0.447
≥1 time per day	89.80% (88)	86.08% (68)	
Snack			
Never/rare	7.14% (7)	22.78% (18)	0.003*
≥1 time per day	92.86% (91)	77.22% (61)	
Lollipop candy			
Never/rare	47.96% (47)	65.82% (52)	0.017*
≥1 time per day	52.04% (51)	34.18% (27)	
Other candy			
Never/rare	33.67% (33)	51.90% (41)	0.015*
≥1 time per day	66.33% (65)	48.10% (38)	
Cakes			
Never/rare	21.43% (21)	11.39% (9)	0.077
≥1 time per day	78.57% (77)	88.61% (70)	
**Child environment**			
Water type			
Bottle water	66.33% (65)	73.42% (58)	0.752
Tap water	14.29% (14)	12.66% (10)	
Well underground water	8.16% (8)	6.33% (5)	
Mixed sources	11.22% (11)	7.59% (6)	
**Child feeding**			
Breast feeding (Y)	97.96% (96)	98.73% (78)	0.581
Bottle feeding (Y)	73.47% (72)	86.08% (68)	0.040*
Bottle fed with sweet milk (Y)	9.18% (9)	2.53% (2)	0.062
**Child oral health status**			
*S. mutans* level			
SM Strip score 0 - CFU <10^4^	9.18% (9)	63.29% (50)	<0.001*
SM strip score 1 – CFU 10^4^ - 10^5^	7.14% (7)	16.46% (13)	<0.001*
SM strip score 2 – CFU 10^5^ - 10^6^	25.51% (25)	12.66% (10)
SM strip score 3 – CFU >10^6^	58.16% (57)	7.59% (6)
Smooth surface caries (number of surfaces, mean ± SD)	4.42 ± 5.6	0	

*Statistical significance level <0.05; Chi-squared test.

### Salivary Microbiome Diversity

**Figure 1:**
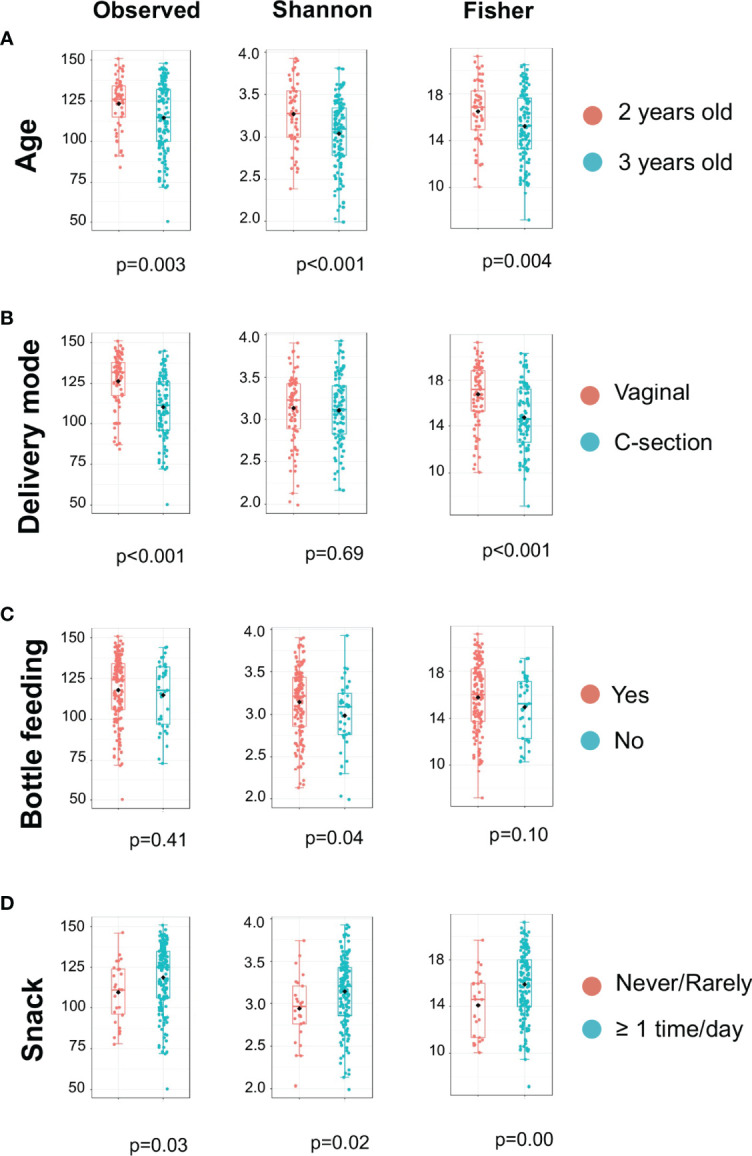
Alpha Diversity of salivary microbiome among children. Microbial variation measured by alpha diversity index among children from different age group **(A)**, mode of delivery **(B)**, Bottle feeding **(C)**, and Consumption of snacks **(D)**. T-test was used for the statistical comparisons.

The microbiome data include 79,001 OTUs [excluding OTUs with fewer than two sequences and sequences that fail to align with PyNAST (Caporaso et al., 2010)] for 354 samples. The sequence reads of all samples in the study are deposited in the NCBI Sequence Read Archive (SRA) as a study under the accession number of PRJNA824062. For the rarefaction curves, see **Figure S1**. Microbial community variation measured by alpha diversity was seen among children from different age groups, the mode of delivery, mother chew food practice, and children’s ECC status (**Figure 1**). For instance, the salivary microbiome of 2-year-old children and born vaginally had a higher alpha diversity (Observed and Fischer index) than 3-year-old children and born with C-section (**Figures 1A, B**) . The alpha diversity was significantly higher in children who frequently consumed snacks (**Figure 1D**).

**Figure 2:**
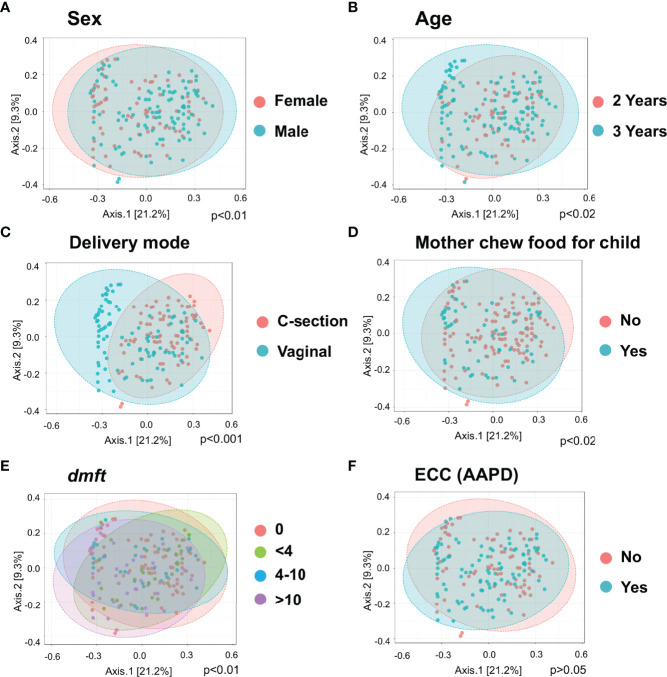
Diversity of salivary microbiome among children. Principle coordinate analysis (PCOA) plot is generated using OTU metrics based on beta diversity (Bray-Curtis index) for different sex groups **(A)**, Age **(B)**, Mode of delivery **(C)**, Mother chew food for child **(D)**, Child caries severity *dmft* **(E)**, and ECC status **(F)**. Permutational MANOVA (PERMANOVA) was used for these statistical comparisons between or among the categorical groups.

Principle coordinate analysis (PCOA) plot was generated using OTU metrics based on beta diversity (Bray-Curtis index). The study demonstrated the beta diversity of the salivary microbiome of children differs depending on sex (p<0.01), age (p=0.02), mode of delivery (p<0.001), whether mother chews food for the child (p=0.02), and children’s caries severity measured by *dmft* (p<0.01) (**Figure 2**). However, the overall beta diversity was not significantly different among ECC and caries-free children (when ECC is defined using AAPD criteria) (**Figure 2F**).

### Salivary Microbiome Core

**Figure 3:**
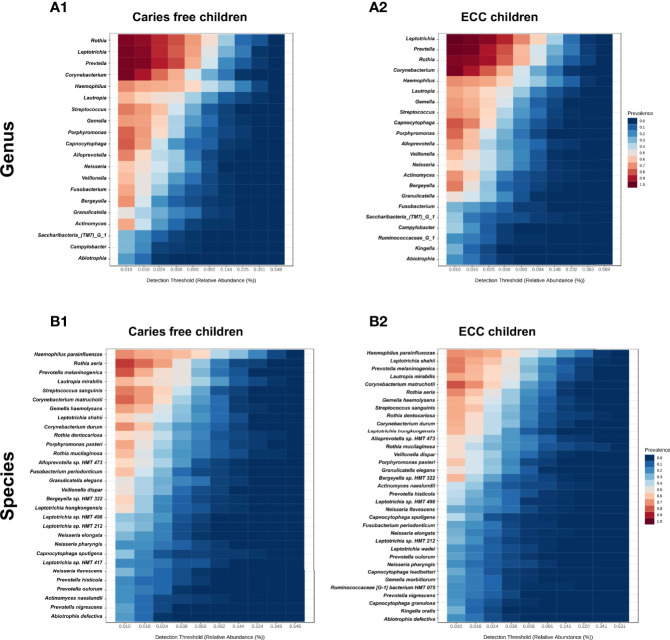
Core salivary microbiome of ECC and carries-free children. Taxa at the genus and species level with more than 20% prevalence and more than 0.01% relative abundance are depicted. in caries free children **(A1, B1)** and ECC children **(A2, B2)**.

Taxa at the genus and species level with more than 20% prevalence and more than 0.01% relative abundance are depicted. Interestingly, most of the genera and species were the same among the ECC and the caries-free children. However, several core taxa relative abundance and prevalence differed between the two groups. For instance, *Rothia aeria, Prevotella melaninogenica, Streptococcus sanguinis*, and *Corynebacterium durum* were more prevalent and enriched in caries-free children, while *Leptotrichia shahii* and *Corynebacterium matruchotii* were more prevalent and enriched in ECC children (**Figure 3**).

### Discriminate Features Between ECC and Caries-Free Children

**Figure 4:**
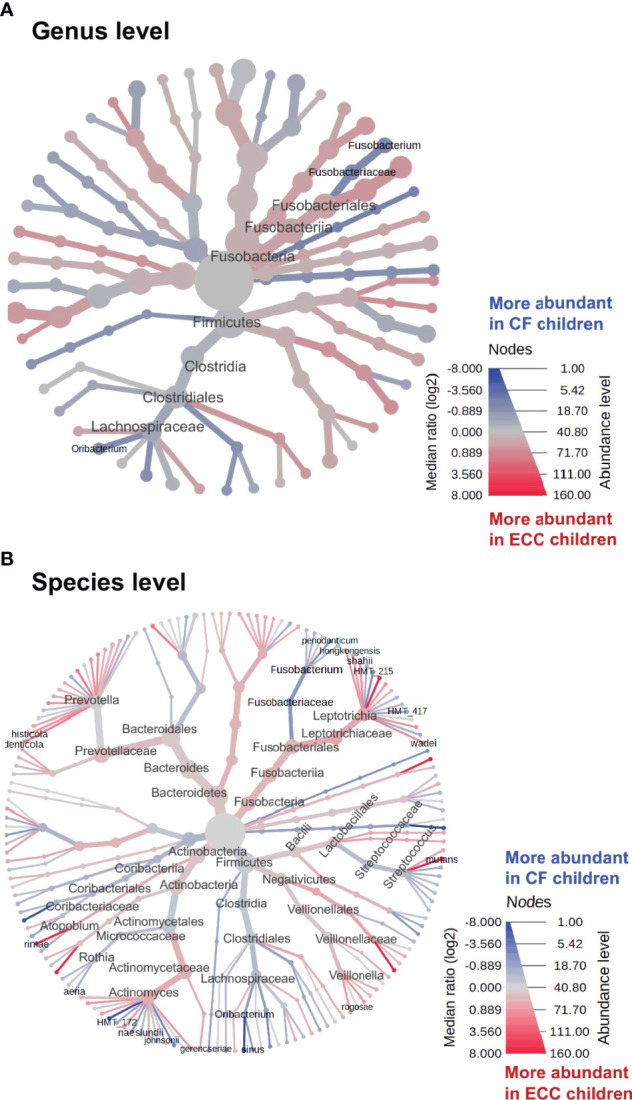
Heat trees of salivary microbiome abundance among ECC and caries-free children. The heat trees depict the OTU classifications and differential abundance comparison at the genus level **(A)** and at the species level **(B)** between salivary microbiome of children with ECC and without ECC. In the heat trees, size and color of nodes and edges are correlated with the abundance ratio of organisms in ECC children vs. caries-free children. Taxa colored in red are enriched in ECC Children, whereas taxa colored in blue are enriched in caries-free children. Taxa with labels indicate a significant abundance difference between ECC children vs. caries-free children measured by the Wilcoxon Rank Sum test (p<0.05).

**Figure 5:**
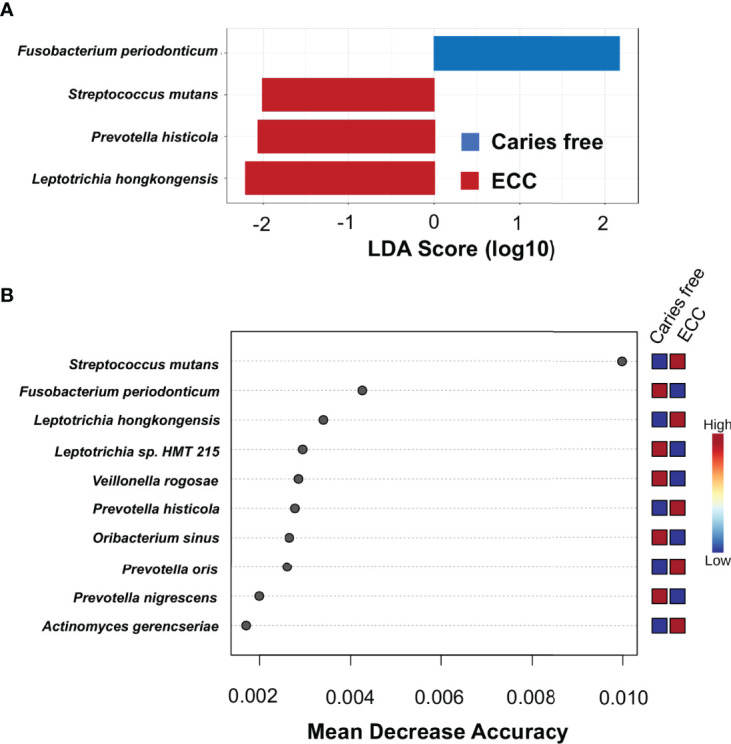
Taxa at genus level differently enriched in ECC and caries-free children. **(A)** Linear discriminant analysis (LDA) effect size (LEfSe) method was performed to compare taxa between ECC and caries-free children. The bar plot lists the significantly differential taxa based on effect size (LDA score log_10_ >2.0 and FDR <0.1). **(B)** Random forest identified important features at the species level that were differently enriched among ECC and caries-free children. Red indicates a higher abundance in ECC, whereas blue indicates a higher abundance in caries-free children.

To identify discriminate taxa between the ECC and caries-free children, heat trees depicted the OTU classifications and differential abundance comparison at the genus level (**Figure 4A**) and at the species level (**Figure 4B**) between the salivary microbiome of children with and without ECC. In the heat trees, size and color of nodes and edges are correlated with the abundance ratio of organisms in ECC children vs. caries-free children. Taxa colored in red are enriched in ECC Children (e.g., *S. mutans*, *P. histicola*, *L. hongkongesis*, and *L. shahii*, etc.). Taxa colored in blue are enriched in caries-free children (e.g., *F. periodonticum*, *Actinomyces gerensceriae*, *Oribacterium sinus*, and *Veillonella rogosae*, etc.). Taxa with labels had a significant differential abundance between ECC children and caries-free children measured by the Wilcoxon Rank Sum test, p<0.05. The significant differential abundances were further verified in the linear discriminant analysis effect size (LEfSe) analysis and random forest at the species level (**Figure 5A**). The ECC group had an increased abundance in *S. mutans*, *P. histicola*, and *L. hongkongesis* compared to an increased abundance in *F. periodonticum, Leptotrichia* sp.*, V. rogosae, O. sinus*, and *P. nigrescens* in caries-free groups (**Figure 5B**).

### ECC Caries Prediction Models

**Figure 6:**
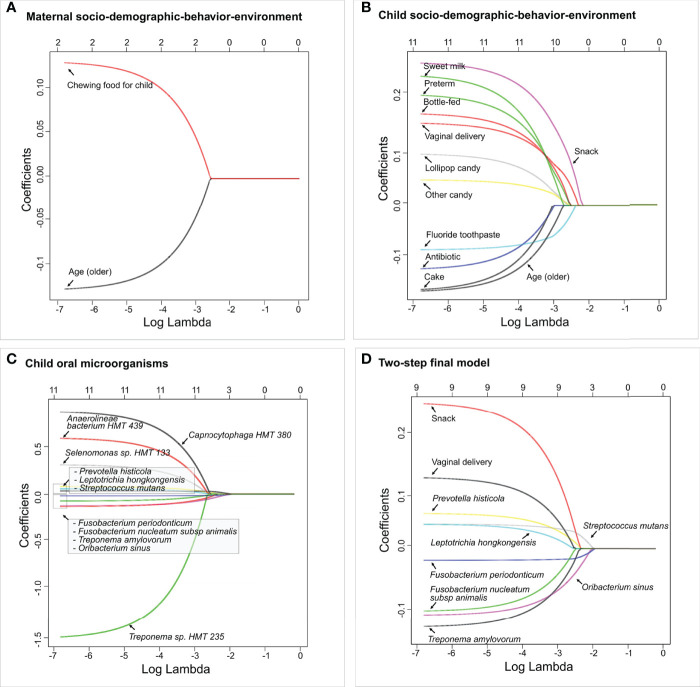
Identified factors associated with child’s caries risk using factors *via* two-step model. LASSO penalized logistic regression modeling was used for caries predictor selection based on 413 variables, including children’s saliva samples. Specifically, variables from three separate platforms were identified shown in **(A)** Maternal socio-demographic-behavior-environmental factors, **(B)** Children’s socio-demographic-behavior-environmental factors, and **(C)** Children’s salivary microorganisms. The final two-step model using variables identified from **(A–C)** is shown in **(D)**. The LASSO solution path above shows how the model is built sequentially by adding one variable at a time to the active set. The 2-step predictive model is the following (area under the curve: 0.85).

All children had more than 100 OTU reads per subject. Ninety OTUs with fewer than 10 reads were removed from the dataset to ensure sufficient depths. The final dataset included 177 children with 416 variables in total, in which seven were maternal socio-demographic-behavior-environmental factors (platform 1), 20 were children’s socio-demographic-behavior-environmental factors (platform 2), and 386 were children’s oral microbial factors (platform 3). In the two-step model building approach, three separate LASSO penalized logistic regression models were fitted for the three platforms in step 1. Two maternal socio-demographic-behavior-environmental factors from the platform 1 (**Figure 6A**), 11 children’s socio-demographic-behavior-environmental factors from the platform 2 (**Figure 6B**), and 11 children’s oral microbial factors from the platform 3 (**Figure 6C**) were selected based on 10-fold cross-validation.

In step 2, the 24 variables from the previous step were used as the candidate variables and the final model selects nine. Nine variables entered the final logistic regression model, they were, in order, *F. periodonticum*, *S. mutans*, *O. sinus*, *P. histicola*, snack, *Treponema amylovorum*, *F. nucleatum* subsp. a*nimalis*, vaginal delivery, and *L. hongkongensis* (**Figure 6D**). In particular, vaginal delivery, snack, *L. hongkongensis*, *P. histicola*, and *S. mutans* had a positive effect on ECC with an increased risk of having caries. *F. nucleatum* subsp. *animalis*, *F. periodonticum*, *O. sinus*, and *T. amylovorum* showed decreased risk for having ECC in our cohort. As a comparison, the one-step model with the same nine variables resulted in the same regression coefficients (**Figure S2**). The area under the curve (AUC) value for the two-step predictive model was 0.85.


$$\begin{array}{cccc} logit\left(p\right)=X\beta =0.283+0.117\text{ vagianl delivery}+0.241\text{ snack} & -0.104\;F.\;nucleatum\;subsp\;animalis-0.019\;F.\;periodonticum & +0.040\;L.\;hongkongensis-0.110\;O.\;sinus+0.058\;P.\;histicola+0.040\;S.\;mutans & -0.130\;T.\;amylovorum \end{array}$$

## Discussion

Caries occurrence in preschool-age children depends on several host and environmental factors, which can ultimately disturb the oral microbiome equilibrium. Studies of caries risk assessment and prediction at an individual level are consequential for a clinical decision-making at a patient-care level and design appropriate evidence-based caries preventive interventions at a community level (Chanpum et al., 2020). The highlight of this study is the interdisciplinary approach incorporating data platforms from multiple sources, which include 1) the carefully acquired mother-child dyads dataset containing information of socio-demographic, medical, and delivery information; caries examination, oral health behavior, and dietary practice; 2) the salivary microbiome 16S rRNA gene sequencing dataset; 3) the use of linear discriminant analysis coupled with effect size (LEfSe) analysis (Segata et al., 2011) to determine differentially abundant bacterial taxa associated with ECC or caries-free children; and 4) the application of a novel machine learning approach to develop a multi-platform caries prediction model with quantifiable coefficients.

**Figure 7:**
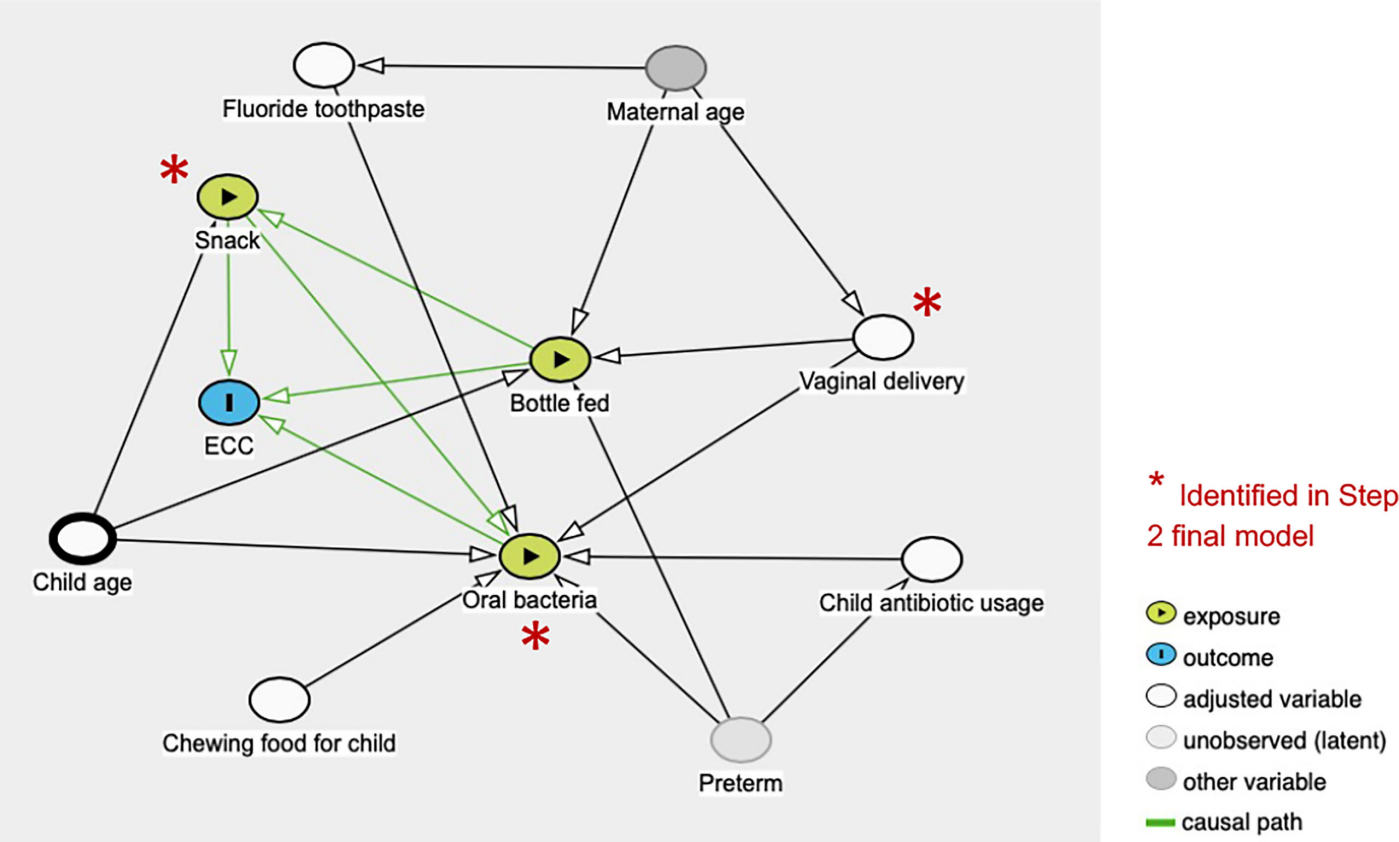
Conceptual causal inference from multiplatform variables assessed in the study.

The initial objective of this study sought to examine the caries-associated risk factors among the 177 mother-child dyads. The significant risk factors for ECC included preterm birth, high levels of *S. mutans*, increased consumption of soft drinks, snacks, and candies, and used less fluoride toothpaste. Vaginal-born children are 2.27 times more likely to experience ECC compared to their counterparts. These findings are consistent with those evidenced in earlier studies (Pattanaporn et al., 2013; Twetman et al., 2020). What is not yet clear is how those plausible caries-associated risk factors impact on oral microbial composition in 2- to 3-year-old children. In addition to microbes, our predictive model was successful at identifying behavioral and environmental risk factors for developing caries; the conceptual inference is drawn in **Figure 7**. Interestingly, our model revealed that vaginal delivery predicted the onset of caries in these Thai children. While there is limited knowledge on how the delivery route impacts the oral ecology of overall oral health, several studies have demonstrated a relationship between the delivery route and oral microbiome development (Xiao et al., 2020). For example, a birth cohort study among Irish infants revealed a higher oral microbial community diversity in children born by cesarean. However, the impact of birth mode on the oral microbiome was only observed up to the first week of age. This influence diminished after the first week of life. Another study examined the oral microbiome of very low birth weight infants and found a higher relative abundance of *Ureaplasma* and *Pantoea* in the vaginal-born infants, but a higher colonization prevalence of *Corynebacterium*, *Methylobacterium*, and *Variovorax* in cesarean-born infants (Li et al., 2020). Other studies indicated that the bacterial profile colonized in infants born vaginally resembles mothers’ vaginal bacterial communities, whereas the microbial community of infants born by cesarean section resembles those present on mothers’ skin (Dominguez-Bello et al., 2010; Lif Holgerson et al., 2011; Drell et al., 2017). Worth noting that research on infants’ gut microbiome development and delivery mode revealed similar findings to the oral microbiome. These findings included an increased similarity to their mother’s gut microbiome when born vaginally (Bäckhed et al., 2015), delayed colonization of prominent commensals in cesarean born infants (Dogra et al., 2015), and lower diversity in cesarean born infants when compared to vaginally-born infants (Hesla et al., 2014). Due to the importance of understanding the impact of the maternal oral microbiome on children’s caries outcome, our future study will further analyze the relatedness of the maternal and children’s salivary bacteriome and their impact on ECC. Importantly, we also found a lower diversity in the cesarean-born children in our study (**Figure 1A**). Further, 3-year-old children had a lower alpha diversity than the children at 2 years of age in our study (**Figure 1B**). This could be reflective of the stabilization of the oral microbiome that occurs after 2 years of age; at this point, children become stably colonized by resident bacteria of the oral cavity and have fewer bacteria that are from environmental exposures (Dashper et al., 2019).

Sugary snacks are known to serve as the substrate for oral microbial metabolism, leading to acidification of the oral environment, favoring aciduric and cariogenic microorganisms, and causing tooth hard tissue demineralization and, ultimately, caries (Marsh, 2018). More than two-thirds (68%) of US 2-year-olds and roughly three-quarters (74%) of 3-year-olds consumed some type of dessert or candy in a day (Fox et al., 2010). Our findings that frequent snacking is a risk factor for caries is supported by previous studies that have emphasized the importance of limiting cariogenic or sugary snack consumption as part of a good oral health routine to prevent caries onset (Hong et al., 2014; Moimaz et al., 2014).

To build the caries-predictive model, the study first identified commonly recognized caries risk microbial factors, such as children’s age, model of delivery, preterm birth, frequency of snack consumption, and presence of *S. mutans* and other potential caries-associated bacteria. Additionally, we detected several other microbial species, either caries-associated or protective that traditionally have received less attention.

For the penalized regression models, both the variables selected into the model and the order they enter the model were important. A variable entering a model sooner (i.e., at a higher lambda value, the tuning parameter) indicates that it is deemed significant under harsher penalties. In our final logistic regression model, the coefficients of vaginal delivery, snack, presence of *S. mutans, L. hongkongensis*, and *P. histicola*, were positive, indicating the change in log-odds of an individual having caries due to a one-unit increase in those variables. More specifically, the coefficient for vaginal delivery in the 2-step model was 0.1174. The odds ratio of having caries for a child who was delivered by vaginal, compared with a child who was c-sectionally delivered, could be 12% higher. Likewise, having snacks increases the odds of having caries by 27%.

Meanwhile, the coefficients of presence of *F. periodonticum, F. nucleatum* subsp. *animalis*, *O. sinus*, and *T. amylovorum* were negative, indicating a decrease in the log-odds of an individual having caries, which means that for a negative coefficient, the subject is less likely to have caries as those variable increase. The results suggest that the enriched levels of those four bacterial species in the saliva present a reduced risk for ECC.

### Predictors Identified in Machine Learning Model

This study identified the plausible caries-promoting microbial species, which were enriched in ECC samples, were *S. mutans, P. histicola*, and *L. hongkongensis.*

### Streptococcus mutans

ECC children are known to have a higher relative abundance of salivary and plaque *S. mutans*, well-known for its acidogenicity, aciduricity, and capability of synthesizing extracellular matrix using carbohydrates (Banas, 2004) (Tanner et al., 2011; Gross et al., 2012; Yang et al., 2012; Ma et al., 2015; Johansson et al., 2016; Richards et al., 2017; Xiao et al., 2018). Not surprisingly, this species was firstly found as caries promoting species in our model.

### Prevotella histicola

*Prevotella histicola* was found in two independent studies (Teng et al., 2015; Wu et al., 2021) that used a machine learning approach to identify discriminative species in caries-active and caries-free children. Despite previously reported association between *P. histicola* and increased caries risk in children (Hurley et al., 2019), it remains unclear about the potential cariogenicity of *P. histocola* and its interaction with other oral microorganisms in leading to a caries-prone condition in the oral cavity.

### Leptotrichia hongkongensis

*Leptotrichia* species normally reside in the oral cavity, gastrointestinal system, and urogenital system. They are typically not considered pathogenic, but may cause opportunistic infections in an immune-suppressed host (Eribe and Olsen, 2017). Xu et al. characterized microbial composition in supragingival plaque samples from children younger than 30 months old and observed that *L. hongkongensis* was identified in caries-affected subjects (Xu et al., 2014). *L. hongkongensis* was also more predominant in adults with active caries (Johansson et al., 2016). Interestingly, *L. hongkongensis* co-occurred with other *Leptotrichia* and *Fusobacterium* species that contributed to black stains of dental caries in primary dentition. Despite the observed associations of *Leptotrichia* species with caries, there remains a gap in understanding their role in the progression of caries.

This study further identified several potentially protective microbial species enriched in caries-free children: *F. periodonticum*, *F. nucleatum* subsp. *animalis, Treponema amylovorum, O. sinus.* Interestingly, three of these species (*F. periodonticum*, *F. nucleatum*, *T. amylovorum*) are commonly known periodontal pathogens.

### Fusobacterium periodonticum

*F. periodonticum*, although without clear evidence on its role in the etiopathogenesis of periodontal disease, is considered to be an opportunistic pathogen residing in deep periodontal pockets and gingival sulcus (Park et al., 2010). Jiang and colleagues observed that *F. periodonticum* was significantly predominant in the saliva of caries-free children compared to caries-affected, which could be suggestive of a caries protective role (Jiang et al., 2016). These findings were also consistent with a similar case-control cohort study examining bacterial profiles of adults with caries (Belstrøm et al., 2014). A more recent study analyzed the variation of the tongue microbiota and its association with dental caries among 6-7-year-old and 11-12-year-old children. The study results demonstrated a more frequent presentation of *F. periodonticum* in children without history of dental caries (Zhang et al., 2021). Thus, salivary counts of *F. periodonticum* can be used as a biomarker and may help caries risk screening (Belstrøm et al., 2014; Jiang et al., 2016).

### Fusobacterium nucleatum

*F. nucleatum* is a commonly recognized periodontal pathogen and regarded as a bridging colonizer that adheres to the early colonizers of dental plaque, followed by adhesion from late plaque colonizers (Aruni et al., 2015). Its FadA adhesin has been identified as a virulence factor for both *F. nucleatum and F. periodonticum*, which is absent in other fusobacterium species and are considered to assist them in binding to host cells (Han, 2015). Studies have reportedly detected *Fusobacterium* species including *F. nucleatum* in abundance in the healthy oral microbiota among children (Teng et al., 2015). Tanner et al. reported *F. nucleatum* as more frequently detected in caries-free children (Tanner et al., 2011). Heinrich-Weltzien et al., indicated that *F. nucleatum* was found more frequently in non-discolored plaque samples (Tanner et al., 2011).

### Treponema amylovorum

*T. amylovorum* species are highly motile, fastidious, saccharolytic gram-negative spirochetes majorly associated with subgingival plaque in periodontal disease (Wyss et al., 1997; Siqueira and Rôças, 2004). Although the majority of the scientific evidence links *T. amylovorum* with periodontal and endodontic diseases, our study results indicated that the presence and higher abundance of *T. amylovorum* in Thai children are associated with a lower risk for ECC, which is a novel finding.

### Oribacterium sinus

*O. sinus* was initially isolated from purulent discharge of a 6-year-old-child with bilateral maxillary sinusitis (Carlier et al., 2004). A longitudinal cohort study examined the oral microbiome development during the first four years of life and subsequent development of ECC (Dashper et al., 2019). Although *O. sinus* had a higher (≥90%) prevalence when children are between 1-4 years of age, making its way to the ‘core oral microbiome’ of young children, the relative abundance of *O. sinus* was not suggested to be significantly associated with caries status (Dashper et al., 2019).

### Veillonella rogosae

*V. rogosae* has been identified as a species associated with caries-free condition from other discriminate methods. *Veillonella* species, including *V. rogosae*, are early plaque colonizers with metabolic requirements that are dependent upon organic acids, including lactic acid, produced by *Streptococcus*. Thus, together these species are considered to co-aggregate and contribute to biofilm formation and maturation in early stages (Mashima and Nakazawa, 2014). Studies have reported that age, geographic location, diet, and oral health behaviors influence the proportion of oral *Veillonella* species. One such study (Djais et al., 2019) examined the proportion of oral *Veillonella* species in the saliva samples of Japanese children 4- to 14-years-old as a measure of their oral health status. Interestingly, the detection of *V. rogosae* declined with deteriorating oral hygiene status (49.1% in good hygiene vs. 44.4% in moderate hygiene vs. 34.1% in poor oral hygiene). A study in Thai children with different oral hygiene status (good, moderate, and poor) revealed similar findings. *V. rogosae* prevalence was significantly lower in the poor oral hygiene group than in the good oral hygiene groups, whereas, *Veillonella parvula* and *Veillonella tobetsuensis* were significantly more prevalent in the poor oral hygiene group (Mashima et al., 2016; Theodorea et al., 2017). These findings, together with ours, suggest that the abundance of *V. rogosae* may be a predictor of good oral hygiene status and potentially a caries protective factor among children.

We took two data integration approaches in this paper. The first parallel integration approach treats factors on different platforms equally and assumes no specific correlation structures among factors. The second hierarchical integration approach is indeed an integration with pre-selection on each platform first. It can be considered to take into account the correlation structure within each platform. The two approaches usually produce different outcomes since the pools of candidate predictor variables are different but are expected to have a great overlap. It is a coincidence that the two models in this paper are exactly the same, which also indicates good reproducibility and reliability.

The following limitations need to be considered when interpreting the study results: 1) The study was conducted in one Thailand city. Thus, generalization to other populations is unreliable due to the difference in racial, ethnical, cultural, and dietary background; 2) With the dataset being cross-sectional, the models are built upon the existing caries status, not through the longitudinal onset of caries. Future validations of our models are warranted using longitudinal dataset. There is an additional need for a more mechanistic understanding of how bacteria, such as *V. rogosae* and *L. hongkongensis*, are involved in protecting the oral cavity from caries progression.

## Conclusions

Multimodal data integration using statistical machine learning models revealed that the mode of delivery and sugary snack consumption outranks salivary microbiome in predicting dental caries in a large cohort of mother-child dyads living in Thailand. Future machine learning approaches that include microbial and environmental risk factors are needed to comprehensively assess the dynamic changes of children’s caries risk factors in a longitudinal cohort.

## Data Availability Statement

The datasets presented in this study can be found in online repositories. The name of the repository and accession number can be found below: NCBI; PRJNA824062.

## Ethics Statement

The Ethical Committee approved the protocol for this study of the Faculty of Dentistry, Chiang Mai University, Thailand (No. 12/2008). Written informed consent to participate in this study was provided by the participants’ legal guardian/next of kin.

## Author Contributions

TTW, JX, and YL contributed to the conception, design, data acquisition, analysis, and interpretation, drafting and critically revising the manuscript. PS and KP contributed to the clinical data collection. PS was responsible for microbial DNA extraction. BP and CG were responsible for DNA sequencing. SM, SV, and YZ contributed to data acquisition, analysis, and critically revising the manuscript. All authors have read and approved the final version of the manuscript and agree to be accountable for all aspects of the work.

## Funding

Dr. JX research was supported by the National Institute of Dental and Craniofacial Research grant K23DE027412.

## Conflict of Interest

The authors declare that the research was conducted in the absence of any commercial or financial relationships that could be construed as a potential conflict of interest.

## Publisher’s Note

All claims expressed in this article are solely those of the authors and do not necessarily represent those of their affiliated organizations, or those of the publisher, the editors and the reviewers. Any product that may be evaluated in this article, or claim that may be made by its manufacturer, is not guaranteed or endorsed by the publisher.
