# Supplementary material for: Multimodal Data Integration Reveals Mode of Delivery and Snack Consumption Outrank Salivary Microbiome in Association With Caries Outcome in Thai Children
Source: Front Cell Infect Microbiol. 2022 May 23;12:881899. doi: 10.3389/fcimb.2022.881899 (PMC9168266; doi:10.3389/fcimb.2022.881899)
Supplement: Supplementary file 1 [file DataSheet_1.docx]

**Supplemental figures**

**Figure S1. Rarefaction curve of children’s salivary oral microbiome**

**Figure S2. Identified factors associated with child’s caries risk using one-step machine Learning model**

The 1-step predictive model is the following (area under the curve: 0.85):

$$logit\left( p \right)=X\beta=0.283+0.117 Vagianl delivery+0.241 snack-0.104 Fusobacterium nucleatum subsp animalis-0.019 Fusobacterium periodonticum+0.040 Leptotrichia hongkongensis-0.110 Oribacterius sinus+0.058 Prevotella histicola+0.040 Streptococcus mutans-0.130 Treponema amylovorum$$
